# A Review on Applications and Uses of *Thymus* in the Food Industry

**DOI:** 10.3390/plants9080961

**Published:** 2020-07-30

**Authors:** Gema Nieto

**Affiliations:** Department of Food Technology, Food Science and Nutrition, Faculty of Veterinary Sciences, Regional Campus of International Excellence “Campus Mare Nostrum”, Espinardo, 30071 Murcia, Spain; gnieto@um.es; Tel.: +34-868-889-624; Fax: +34-868-884-147

**Keywords:** antioxidant, antimicrobial, thyme, phenolic compounds, food additives, food preservatives

## Abstract

Thyme is one of the most important medicinal plants because of its ethnopharmacological relevance and high content of bioactive compounds. This review focuses particularly on thyme as an alternative natural antioxidant and antimicrobial with potential use in the food industry. This is in line with the preferences of the current consumer, who demands healthier and more natural products. Different studies have concluded that the use of thyme increases stability and reduces lipid oxidation during the shelf-life period of foods (meat, meat products, milk, fish or fish products), which makes thyme a promising source of natural additives. Despite these findings, the use of *Thymus* extracts or essential oils as natural additives in foods is reduced in comparison with other natural preservative extracts. This review provides an overview of the most important information on the positive effect of the bioactive compounds of thyme and its uses as a preservative in foods, taking into account its origin (from plants, plant extracts or essential oils).

## 1. Introduction

Plants produce phytochemicals to protect themselves from bacteria, viruses and fungi, but they also protect food from spoilage when added to food. In recent years, great interest has been focused on using natural preservatives in food products in light of recent studies that have indicated the possible adverse effects related to the consumption of synthetic additives. In addition, natural preservatives improve human health because they protect against diseases [1].

Currently, the natural plant extract industry is moving millions of euros around the world. Approximately 1340 plants are known to be potential sources of antioxidant and antimicrobial components [2], and more than 250,000 plant species contain a wide variety of bioactive components. In 1999 alone, the global business of selling natural supplements exceeded 15 trillion dollars, of which $7 trillion was in Europe and $3 billion was in North America; and every year, the sales increase. Plant extracts are included in the group of additives classified as “aromatic and flavoring substances”, which include “all natural products and corresponding synthetic products”, and can be consumed by all animal species without any restriction on the dose of product. Since these products are highly acceptable to the consumer, they are one of the future potential alternatives to synthetic preservatives, and the search for new substances represents an important area of research in the field of food additives. All this has led to the use of natural preservative substances, either directly added to food commodities or incorporated into the diet of the supply species.

Herbs and spices are among the natural compounds that are currently being used as food preservatives, which contain compounds with marked antioxidant and antimicrobial properties [3]. Plant extracts have been used for hundreds of years to improve the organoleptic properties of food, but further to this, Chipault et al. [4,5] reported that plant extracts also have preservative properties in different types of spices, and there is currently a lot of information about the compounds and mechanisms involved in the inhibition of lipid peroxidation [6,7]. They are considered to be GRAS (generally recognized as safe), which makes consumers and regulatory agencies regard them as more appropriate for use in food than artificial compounds. Examples of these synthetic preservatives are butylated hydroxyanisole (BHA) and butylated hydroxytoluene (BHT), which have been used for years in the food industry. However, there is abundant information that indicates that these compounds are potentially carcinogenic, which has generated a growing interest in alternative products that protect against oxidation but are more natural [8,9].

Material plants, such as herbs of the Labiatae family, have been some of the most studied for their preservative properties [10]. Together with their antimicrobial and antioxidant activity, natural extracts have other applications, such as anti-inflammatory, immunomodulatory, spasmolytic and sedative purposes. Natural extracts often owe their biological activity to the synergism between their various compounds since their separate activities are much lower than their combined activity. The toxicity of extracts is considered to be lower when all their components are as found than when they are purified, which is a phenomenon called buffering. As indicated above, food companies are currently showing an increasing interest in the incorporation of natural antioxidants and antimicrobials into food in response to the growing demand by consumers for safer and more natural foods and their rejection of the incorporation of synthetic antioxidants, which are being reassessed for the possible toxicity and carcinogenicity of the components that are formed during their degradation.

In the last decade, a series of investigations have been carried out in order to identify natural substances that are capable of inhibiting lipid autooxidation reactions in meat products and precooked meats after being added during processing operations [11]. Special emphasis has been placed on thyme, rosemary and oregano for their substantial antioxidant activity [12]. However, it is necessary to take into account that the effectiveness of plant extracts depends on the type of substrate on which they act [5,6] and on the administered dose. As a general rule, antioxidants extracted from plants tend to show prooxidant activity at low concentrations and antioxidant activity above a critical concentration [13].

There are several important classes of thyme, such as *Thyme vulgaris*, also called common thyme, which belongs to the Lamiaceae family, and it is native to Mediterranean countries in Europe. In addition, *Thymus zygis* subsp. *gracilis* grows in southern Spain and northern Africa, although it is mainly represented in Spanish regions, such as east of Andalucia, Albacete and Murcia, and is widely used as a condiment. It is a woody-based herb with aromatic pink flowers and green leaves. This spice is the most commercialized in Spain, where it is distilled to extract its essential oil and also exploited as the “thyme leaf” to obtain natural additives for the food industry. It is highly valuable as a flavoring agent with a spicy taste and commonly added to rabbit, boar and lamb meats. In traditional medicine, thyme has been used as a sedative, a carminative, an additive for baths or an infusion for the treatment of skin diseases [14]. From a commercial point of view, the components that have the highest economic interest and impart quality to the essential oil of these plants are thymol (68.1%) and carvacrol (10%), which constitute the majority of and most active compounds [15]. However, thyme essential oil (EO) contains more than 60 ingredients, most of which have important beneficial effects, including antiseptic, carminative, antioxidant, antimicrobial and anticancer properties.

Thyme contains monoterpene phenols, including carvacrol, thymol and p-cymene, and other monoterpenes, such as α-pinene, 1,8-cineole, camphor, linalool and borneol [16].

Taking into account that thyme has been extensively studied for its antioxidant and antimicrobial activities with the target of improving the quality of food, the objective of this article is to review the natural preservative properties of thyme and its mechanisms of action against lipid oxidation and microorganisms. In addition, factors that interfere with their forms of application and effectiveness, interactions and synergistic effects are described.

## 2. Thyme

Thyme is an aromatic plant and is widely distributed over the Mediterranean area (Europe, Asia and North Africa). Taxonomically, thyme belongs to the family of the Labiatae (*Lamiaceae*), the genus *Thymus* (etymologically from the Latin «Thymún» and from the Greek «Thymon») and to the class of Dicotyledons, native to the countries of the western Mediterranean basin. Thyme is one of the medicinal aromatic plants found in the Iberian Peninsula, and its essential oil has become one of the most widely used in the food industry. Thyme has been used since ancient times for its health properties, which are associated with its essential oils and chemical components. Its economic importance is associated with its essential oils.

The generic name comes from the Greek verb Thym, which translates to perfume, in allusion to the intense and pleasant aroma of the plant. It is an aromatic, vivacious, woody, very polymorphic plant that is 10–40 cm high, with numerous branches that are woody, erect, compact, and brownish or velvety-white. The linear, oblong leaves are 3–8 mm, with the petiole or its margins ciliated and whitish on its underside. The flowers are axillary and grouped at the tip of the branches, forming a kind of terminal node. The fruit is a tetraquenium and brown in color. It blooms from March onwards. It is a highly variable species, both in its phenology and in the chemical composition of its essential oil, in which seven chemotypes have already been detected. For this reason, there is often taxonomic confusion in this genus, the varieties or ecotypes of which have been identified as different species. Its habitat is in the countries of the western Mediterranean Basin in dry and sunny soils. It is abundant in the east, center and south of the Iberian Peninsula. It develops in limy, clayey and, less frequently, siliceous soils. It grows at an altitude of 0–1800 m. The climate is warm-temperate and mountainous. It is resistant to frost and drought, but not waterlogging or excess environmental humidity.

**Figure 1:**
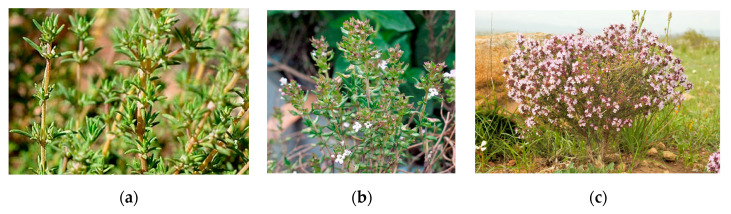
*Thymus zygis* subsp. *gracilis* (**a**); *Thymus vulgaris* (**b**); *Thymus hyemalis* (**c**).

The number of species currently cataloged exceeds 500, although there are perhaps many more that exist because of, among other things, the great ease in which this aromatic plant produces hybridizations and mutations. Among the best-known species in Spain that experience greater propagation and exploitation are *Thymus rumidicus hispánicos*, *Thymus zygis*, *Thymus vulgaris*, *Thymus hyemalis*, *Thymus mastichina*, *Thymus citríodotus*, *Thymus corydothymus*, *Thymus loscossi*, *Thymus pipirella*, *Thymus communis*, etc. (Figure 1).

In all thyme species and varieties, the main part used commercially is its leaves, with purposes that vary from seasoning to herbalism. Another important use that mainly involves the species *Thymus zygis, Thymus mastichina, Thymus corydothymus* and some others is the extraction of essential oils through the distillation process. The essence of thyme has multiple applications, both in medicine and perfumery. From the essence of thyme, balsamic, vermicidal and bactericidal substances are extracted for very diverse uses.

## 3. Composition of Thyme (from Plants, Plant Extracts or Essential Oils)

Several studies have reported that thyme is a source rich in bioactive compounds. Among the phenolic components of thyme oil are thymol and carvacrol, the latter of which is an isomer of the former. Thymol provides thyme oil with its olfactory peculiarities. Depending on the place of origin and the species of thyme, this oil offers percentages of phenolic content that range from 40 to 80 percent of thymol and up to 55 percent of carvacrol.

Rota et al. [15] studied the variability in the essential oil content of *Thymus zygis* subsp. *gracilis*, and of more than 60 bioactive compounds, the most common were thymol and linalool. The in vitro antioxidant activities of essential oils and thyme extracts were reported by Tepe et al. [17].

Thyme contains monoterpene phenols, including carvacrol (iso-propyl-ortho-cresol; 0.4–20.6%), thymol (2-isopropyl-5-methylphenol or iso-propylmeta-cresol; C10H14O; 10–64%) and p-cymene (9.1–22.2%), and other monoterpenes, such as α-pinene (0.9–6.6%), 1,8-cineole (0.2–14.2%), camphor (0–7.3%), linalool (2.2–4.8%) and borneol (0.6–7.5%) [16].

The composition of *Thymus* essential oil is generally a mixture of monoterpenes, which generally contains 10% carvacrol and approximately 50% thymol. The essential oil of thyme is also a source of linalool, α-terpineol, camphor, caryophyllene and γ-terpinene [14]. In addition, it has been reported that methanolic extracts of thyme are sources of flavonols, such as quercetin-7-O-glucoside, and phenolic acids (p-coumaric, caffeic, rosmarinic, cinnamic, carnosic, ferulic, quinic acids and caffeoylquinic), as well as flavanones (naringenin) and flavones (apigenin) [18]. Using other solvents, such as butanol, ethyl acetate and hexane, other compounds can be extracted from thyme, including saponins, steroids, flavonoids, alkaloids and tannins [18].

Of the major compounds in thyme, the content of thymol in Thymus zygis and Thymus vulgaris amounts to 22.3–43.3%and 38.1 %, respectively. In addition, carvacrol accounts for 2.3%, along with other prevailing compounds such as p-cymene (29.1%), g-terpinene (5.2%) and linalool (3.7%) [19]. The chemical composition of the tested thyme is shown in Table 1.

It is important to consider the main factors that influence the effectiveness of natural extracts and essential oils; for example, the biotic characteristics of the plants, such as the season and geographical source, have an influence on the concentration and composition of the bioactive compounds [20]. In addition, the extraction method and the part of the plant used in the extraction (roots, seeds or leaves) also have an important influence on the bioactive compound profile in natural extracts and essential oils [21]. Table 2 reported the functional properties of the main components in thyme.

## 4. Antioxidant Activity of Thyme

In the Labiatae family, thymol is always accompanied by its carvacrol isomer, and both are constituents of the most studied thyme essential oil in terms of its antioxidant activity. Yanishlieva et al. [34] compared the effectiveness of the two components, concluding that during the oxidation of lipids at room temperature, thymol is a better antioxidant than carvacrol, which may be due to the different position of the phenolic group in thymol with respect to that in carvacrol. As previously stated, the chemical composition of essential oils dictates their activity. Tepe et al. [17] analyzed the antioxidant behavior of two varieties of *Thymus sipyleus*, and they reported different constituents in their essential oil since, in one of them, mostly alcohols such as borneol (11.2%) were identified, while the other variety was characterized by the presence of carvacrol (58.1%) and thymol (20.5%). The authors concluded that phenolic oils were more effective in all tests performed.

In general, the inhibitory capacity of oxidation presented by thyme extracts is fundamentally due to its richness in phenolic compounds [11]. The antioxidant activity of these compounds has been evidenced by various authors and is related to their ability to act as scavengers of free radicals, metal ion chelators and inhibitors of oxidative enzymes [35]. Technological and instrumental advances have made it possible to identify and quantify the antioxidant activity of most of the phenolic compounds that exist in plant extracts [36]. However, the antioxidant activity of polyphenolic compounds is dependent on the method used for their extraction. A large number of fractionation and distillation techniques using organic solvents are reported in the literature [37]. In this context, Chang et al. [38] concluded that the effectiveness of the extract depends on the solvent used in distillation. For this reason, methanol has been the most commonly used solvent for obtaining commercial extracts [37]. Initially, alterations in flavor derived from the addition of spices and plants (oregano, thyme, marjoram, rosemary, green tea, sage, etc.) to foods considerably limited their use as antioxidant additives. However, as a solution to this problem, it is possible to introduce a series of stages of deodorization into the process. Currently, the optimization of extraction, purification and fractionation methods has allowed the production of a wide range of extracts that have refined standardized activity and are flavor-free. According to the body of literature, many researchers advise the use of plant extracts because they are effective, economical and considered to be GRAS.

Phenolic constituents appear to be primarily responsible for the antioxidant properties of these substances. However, Dorman et al. [39] established that there is not a direct relation between the antioxidant effectiveness of an extract and its total phenolic compound content. However, Soares et al. [40] evaluated the effectiveness of antioxidants from concentrates obtained from *Thymus zygis* and determined that there is an apparent relationship between the antioxidant potential of these extracts and the total phenols that they contain. One of the sources of the antioxidant properties of the phenols in thyme is their ability to exhibit redox properties and neutralize free radicals [41].

The antioxidant activity of thyme has been demonstrated through several in vitro and in vivo methods. Using in vitro methods, Aouam et al. [42] reported that *Thymus riatarum* showed a high DPPH radical-scavenging capacity and ferric reducing power in ethanolic, aqueous and methanolic extracts. In addition, Golkar et al. [43] showed the cytotoxic activities of phenolic compounds in the essential oil of thyme by using a phosphomolybdate assay.

In a comparison of the antioxidant activity between thyme and other antioxidant compounds, thyme was reported to be one of the best antioxidants. Focusing first on the mechanism of the antioxidant activity of thyme, Lee and Shibamoto [44] conducted a study on the antioxidant activity of volatile components from several plants, including thyme, which produced better results, with an inhibitory effect similar to that of α-tocopherol or BHT (butylhydroxytoluene). In addition, Schwarz and Ernst [45] published an interesting study in which the antioxidant compounds present in different species of thyme were analyzed, among which was *Thymus vulgaris*. These authors identified a new component, p-cimen-2,3-diol, isolated for the first time from thyme leaves, which showed higher antioxidant activity than that of α-tocopherol and BHA (butylhydroxyanisole). Moreover, Youdim et al. [46] carried out a study on the antioxidant properties of the essential oil of *Thymus zygis* and its major components, confirming that the effectiveness of these substances increased in the following order:

Essential oil > thymol > carvacrol > Υ-terpinene > mircene > linalool > ρ-cimene > limonene > 1,8-cineool > α-pinene.

According to these ranks, all the substances tested have antioxidant activity, although, as can be seen from the work of the abovementioned authors, no single component is more effective than the essential oil as a whole.

The antioxidant effect is due to the fact that thyme EO contains at least 60 bioactive compounds with powerful antioxidant properties. The main phenolic compounds are carvacrol (3.5%), thymol (68.1%), ρ-cimeno monoterpene hydrocarbons (11.2%) and γ-terpinen (4.8%) [15], which have significant antioxidant properties. Since poly(phenolic) compounds (as we have indicated on numerous occasions) exhibit redox activity and can act as hydrogen donors, as reducing agents, they also have the ability to chelate metals.

The antioxidant effect of thyme extract has been analyzed in various studies, such as that carried out by Haraguchi et al. [47], who verified the properties of *Thymus vulgaris* extract as a protector against the oxidation of the lipids present in biological membranes, identifying components in the extract with substantial antioxidant power. Moreover, flavonoids that have been isolated from the concentrate of this plant show comparable activity to that of BHT, α-tocopherol or L-ascorbic acid [48]. In the study conducted by Venskutonis et al. [49], it was shown that thyme extract is an effective inhibitor of xanthine oxidase, an enzyme in the group of oxidized reductases that produces oxygen free radicals.

Gavaric et al. [50] reported that thymol is more active in vitro than butylated hydroxytoluene (one of the most important synthetic antioxidants). Thymol and thyme essential oil and thymol had IC50 values of 70.06 and 0.24 μg/mL, respectively, whereas that of BHT was 6.95 μg/mL.

Furthermore, Nagoor Meeran et al. [51] studied the antioxidant properties of thymol, and they concluded that it produces phenoxyl radicals, major transient species and scavengers of hydroxyl free radicals.

In a study that compared thymol with other terpenes, such as limonene, menthone, linalool, caryophyllene, camphor, caryophyllene, menthol and pulegone, Salgado-Garciglia et al. [52] reported that thymol showed the highest antioxidant capacity in three different antioxidant methods in vitro (ferric reducing antioxidant power, DPPH and 2,2′-azino-bis(3-ethylbenzothiazoline-6-sulfonic acid)). However, it has also been found in vitro that thymol may exert prooxidant activities, depending on the concentrations tested. This behavior of thyme was studied in intestinal Caco-2 cells by Llana-Ruiz-Cabello et al. [53].

Using in vivo methods, it has been proven, for example, that supplementing the diet of rats with thyme essential oil (*Thymus vulgaris*) helps these animals retain a favorable antioxidant capacity throughout their lives [54]. In another work, the same authors observed that the daily ingestion of this oil (42.5 mg/kg of weight/day) led to the maintenance of high levels of polyunsaturated fatty acids in rat tissues [55]. Thymol is the main constituent of the essential oil used in this study, with a relative concentration of 49%. However, when administered alone, this phenolic compound does not have a significantly greater effect, which seems to indicate that there are other components in the essential oil that also contribute to its antioxidant activity, a fact that was confirmed by subsequent work, such as the study by Youdim et al. [46] and other studies with other plant extracts that have thymol as a major component [56].

The positive result of incorporating essential oils into the diet had been noted previously [57], in a study that reported an increase in the content of polyunsaturated fatty acids and key enzymes in lipid metabolism in treated animals. Since the amounts of these components decrease as the animal ages, oils from plants such as thyme that are capable of reversing this trend could be of great interest in medicine.

On the other hand, when discussing the properties of thyme, it should be mentioned that, in addition, the essential oil of thyme possesses non-volatile chemical components that have equally interesting qualities. These compounds are extracted either directly from the plant or from previously distilled plant material using appropriate solvents and subsequently concentrating the solution obtained. The extracts of these plants are rich in polyphenolic compounds, which have been shown to have antioxidant and antimutagenic activity [17]. For example, it has been proven that luteolin, a flavone constituent of *Thymus vulgaris* extracts, suppresses the mutagenic action of the Trp-P-2 carcinogen, which is formed during food cooking procedures.

Moreover, thymol increases the total antioxidant status in vivo [50] because it boosts the activity of antioxidant enzymes, such as glutathione peroxidase, superoxide dismutase, glutathione-S-transferase and catalase, and the level of other non-enzymatic antioxidants, such as reduced glutathione, vitamin E and vitamin C [58].

## 5. Antimicrobial Activity of Thyme

Essential oils that are rich in phenolic compounds appear to be the most effective compounds against infections caused by microorganisms. As reported above, thyme essential oil is a “natural” preservative with the ability to control microorganisms [59]. It has been proven that there is a synergistic effect between different compounds of the EO, as occurs, for example, between carvacrol and its precursor p-cymene [60]. In addition, it is also necessary to take into account other constituents of these essential oils for their possible antagonistic or synergistic effects, especially thymol and carvacrol [57]. The physical conditions that improve the performance of these oils are low temperature, low oxygen levels and low pH [16]. Anaerobic environments favor the action of both thyme EO and thymol against microorganisms such as *Salmonella thyphimurium* or *Staphylococcus aureus* [61]. On the other hand, Gram-positive bacteria appear to be slightly more sensitive to the action of EO than Gram-negative bacteria [16].

Due to the interaction between the compounds of the EO and the constituents of food, the antimicrobial efficacy of these substances is reduced when they are used in food products, so a higher concentration of essential oil is necessary to achieve results similar to those obtained in vitro [62]. In a study conducted by Shapiro and Guggenheim [63], with bacteria that affect the oral cavity, it was observed that thymol produces a perforation in the plasma membrane of the bacterial cell, which causes a rapid outflow of intracellular constituents. This compound induces a decrease in intracellular ATP as a direct consequence of infiltration, and, in some bacteria, it also inhibits the synthesis pathways of this biomolecule. The effects of thymol on the potential of membranes are probably the result of the infiltration of substances caused by this compound. Evans and Martín [64] also proved the effectiveness of thymol as a growth inhibitor of ruminal microorganisms, such as *Streptococcus bovis* or *Selenomonas ruminantium*.

In fact, thyme has also demonstrated its potential antibacterial activity in vitro against food pathogens, such as *Salmonella, Staphylococcus*, *E. coli*, *Klebsiella*, *Pseudomonas* and *Enterococcus*, at concentrations from 5 to 20 µL EO [65,66].

Indeed, the antifungal, antibacterial, antiparasitic and antiviral activities of thyme plants can be related to their expectorant, anti-inflammatory, antitussive, analgesic, sedative and anti-broncholitic properties.

**Figure 2:**
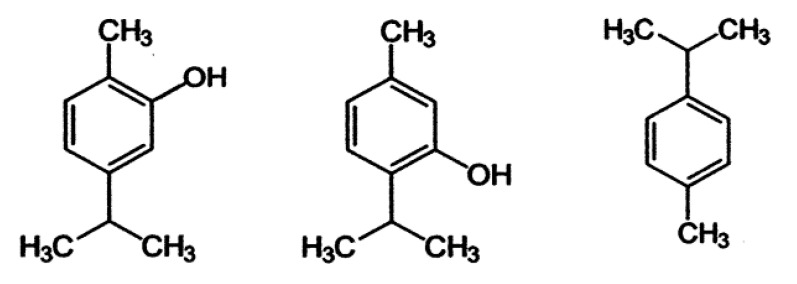
Main chemical structures of terpenes (thymol, carvacrol and p-cymene) found in essential oil of thyme.

On the other hand, carvacrol and thymol are isomeric phenolic compounds that can act against the pathogen *Bacillus cereus* [67]. Carvacrol shows a marked hydrophobic character, so it accumulates in the plasma membrane of the bacterial cell, which, as with thymol, affects its integrity and causes a fall in the membrane potential. In their study, Ultee et al. [67] reported that carvacrol acted to reduce the pH across the plasma membrane, acting as a proton exchanger. This compound, which has a hydroxyl radical in the ortho position (Figure 2), diffuses through the membrane into the cytoplasm of the cell, where it releases its proton. Subsequently, it returns to the cellular membrane to carry a potassium ion from the cytoplasm. The cation is released, and carvacrol captures a new proton, repeating the cycle. The result is the depletion of ATP deposits in the cell, which leads to a deterioration of vital processes and ultimately to the death of the bacteria. Therefore, thymol (with the hydroxyl radical located in the meta position) and carvacrol have strong antibacterial activity. However, ρ-cimeno, the biological precursor of these two constituents in the essential oil of thyme, lacks a hydroxyl group and shows less activity, which suggests that this radical is related to toxicity against microorganisms. This work also showed a synergistic effect between carvacrol and ρ-cimeno, which may be due to the fact that this precursor contributes to the destabilization of the bacterial plasma membrane, which favors the entry of carvacrol into the cell.

Thymol and carvacrol are also active against bacteria such as *P. aeruginosa* or *S. aureus* [68]. These components show an additive effect that causes the inhibition of the growth of these microorganisms by damaging the integrity of the plasma membrane, affecting the pH and the balance of inorganic ions. Di Pascua et al. [69] investigated the bactericidal and bacteriostatic activity of essential oils obtained from various plants on bacteria such as *E. coli*, *S. typhimurium*, *L. monocytogenes* and lactic acid bacteria, and thyme oil was found to be the most effective spice against the greatest number of microorganisms tested. Di Pascua et al. [69] showed the lipophilic nature of thymol and carvacrol, as well as other constituents that can be found in thyme oil, such as limonene or eugenol, and reported that these molecules interact with bacterial membranes, altering their structure and making them more permeable.

Other authors have shown the effectiveness of thyme on *Escherichia coli*. Both the EO obtained from *Thymus vulgaris* and that originating from *Origanum vulgare* exert a strong action on this microorganism, which is observed in a wide range of temperatures [69]. For example, Burt et al. [70] analyzed the effect of EOs of thyme and oregano and their four major components (thymol, carvacrol, ρ-cimeno and γ-terpinene) on *E. coli*, and they proved that thymol and carvacrol have clear bactericidal activity, reporting that they were similarly effective against *E. coli*. The antimicrobial activity of the EO depends on these two components, which have an additive effect and are not influenced, in this case, by the other major constituents of oil, namely, the precursors ρ-cimeno and γ-terpinene, which apparently do not act against this bacterium. The absence of synergism between carvacrol and ρ-cimeno contrasts with that expressed by other authors [71]. This could be due to the physiological differences between the bacteria used in each of the studies since the structure of the cell wall of *Escherichia coli* and other Gram-negative bacteria can inhibit the action of ρ-cimeno. As in previous studies, it was also demonstrated that thymol destroys the integrity and affects the electrical potential of the plasma membrane of the bacterial cell of *E. coli*, which finally leads to cell lysis [72].

Investigating the activity of various antimicrobial agents obtained from plants, Dorman and Deans [73] determined that the EO of thyme has a greater spectrum of action, and, by studying these components separately, these authors confirmed the greater effectiveness of the phenolic compounds present in these oils, especially thymol and carvacrol, with ρ-cimeno being the least active constituent. This test also shows the influence of the hydroxyl group in the phenolic structure since a great difference can be seen between the antibacterial activity of carvacrol and that of its methyl ester, which is a relatively inefficient component; the authors also noted the importance of the position of this group in the benzene ring, contrary to what was previously reflected in this section. They agreed, however, with what was stated by Domingo and López-Brea [74], who affirmed that the presence of hydroxyl groups in phenolics is related to the toxicity of these compounds, and the position of these radicals influences the effectiveness of phenols against bacteria.

In addition, *Thymus albicans* and *Thymus mastichina*, with 1,8-cineole as the main constituent, also have positive inhibitory effects on bacteria, such as *L. monocytogenes*, *St. aureus* or *Salmonella* sp. [75]. Similarly, the terminal alcohol linalool is active against bacteria of the genus *Leishmania* [76] and other microorganisms such as *Lactobacillus plantarum*, *Citrobacter freundii* or *Clostridium sporogenes* [73]. Alcohols have more bactericidal than bacteriostatic activity, as they denature the proteins of microorganisms [77]. In summary, essential oils are effective against a wide variety of microorganisms and can be used as food preservatives when added in small amounts; they can delay microbiological contamination and food deterioration without affecting its organoleptic properties [73]. Moreover, baicalein, another flavone identified in the *Thymus vulgaris* extract, potentiates the antimicrobial effect of tetracycline on *S. aureus* [78]. Other studies have reported the antibacterial potential of different plant extracts (thyme, fennel, sage, tea and mint) and determined that thyme is the most effective against common pathogenic bacteria and lactic acid bacteria, so these concentrates are considered natural foods or food additives that could have a positive effect on the digestive system of humans and animals [79]. The aqueous extract obtained from *Thymus vulgaris* was screened and found to be one of the most effective against bacteria, such as *H. pylori* [80]. The ability of compounds to act against different microorganisms from the extracts of other thyme species, such as *Thymus serpyllum* [81] or *Thymus spathulifolius* [82], has also been successfully tested. In the latter article, the authors compared the effects of the EO of the plant, containing thymol (36.5%) and carvacrol (29.8%) as the major constituents, with those of the extract. The result showed the strong antimicrobial activity of the EO, both against bacteria and against most of the fungal species tested by these authors, while the extract acted moderately against bacteria and was not effective against fungi. In other work, the antioxidant capacity of these two substances was evaluated, with positive results for both [15].

## 6. Thyme as Functional Food

The use of herbs and spices dates back to 5000 B.C. Therefore, they might be considered one of the first functional foods. Experimental evidence supports the health benefits attributed to spices and herbs, for example, cardio-protective and anti-atherogenic potential, digestive stimulant action, antidiabetic effects, antilithogenic properties, cancer-preventive potential and anti-inflammatory properties [83,84,85].

Nowadays, spices are considered by the scientific community to be potential providers of health benefits beyond only food adjuncts for flavor and taste.

Although there is not only one definition of functional food, the following definition: “Natural or processed foods that contain known or unknown biologically-active compounds; which, in defined, effective non-toxic amounts, provide a clinically proven and documented health benefit for the prevention, management, or treatment of chronic disease”. Therefore, thyme can be considered a functional food due to the provided benefits other than basic nutrition. Herbs and spices have been used for their health properties and preservative properties in various cultures, such as in traditional Indian or Mediterranean cuisine (with the combination of herbs, spices and other foods). These kinds of diets are related to lower incidence rates of chronic diseases, including certain forms of cardiovascular disease and cancer, and these effects are due to the synergistic and the additive effects of the complex mixture of the bioactive compounds contained in fruits, vegetables, spices and herbs. Western societies tend to focus on a single food instead of whole dishes. A diet rich in spices not only enhances the taste of food but also makes it healthier.

Infusions of thyme have been used for their health benefits for thousands of years as a cold treatment, antispasmodic, a carminative agent, traditional medicine in digestive problems and an expectorant for upper respiratory tract infections. In addition, the potential therapeutic effects of thymol have been used for the treatment of disorders affecting different organ systems, such as the cardiovascular, nervous, and respiratory systems [83,84,85].

### Potential Health Benefits of Thyme

In general, phenolic compounds have an influence on cardiovascular disease because they reduce molecular damage, prevent plaque formation and inhibit the oxidation of cholesterol. Several researchers have also reported the beneficial health effects of thyme on cardiovascular disease. Kensara et al. [86] reported that the administration of the aqueous extract of *Thyme vulgaris* at 100 mg/kg/day for 8 weeks significantly reduced blood pressure in rats, which exhibited improvements in hypertension and aortic vascular damage. Moreover, Haque et al. [86] studied the oral administration of thymol at a dose of 14 mg/kg twice per day to obese rats (induced with a high-fat diet for 4 weeks), and as result of this administration, leptin and insulin levels improved, lipid levels were lowered, the antioxidant potential was enhanced and visceral fat accumulation was attenuated. In addition, another study with rabbits that were fed a high-fat diet and administered 3 or 6 mg thymol/kg per day reported the lowering of serum lipids, inhibition of inflammation and oxidative stress and suppression of the progression of atherosclerosis or hyperlipidemia [83]. Moreover, bioactive compounds, such as thymol also exhibit anti-inflammatory, anti-carcinogenesis and immunomodulatory properties [87,88,89].

Thymol, which contributes to the antioxidant activity of thyme, is considered an important antioxidant with therapeutic properties. In general, the bioactive components of spices that have shown potent cancer-preventive, anti-inflammatory, anti-atherogenic and antimutagenic bioactivities are in fact antioxidants, which is mainly because these phenolic compounds have a beneficial role in preventing diseases mediated by oxidative stress. For example, several studies have suggested that thymol inhibits lipid peroxidation [90] and the formation of undesirable compounds related to oxidative deterioration.

The ability of thyme to act as an anti-inflammatory agent has been reported in several in vitro and in vivo studies. This herb improved the activity of the enzyme superoxide dismutase (which acts as an anti-inflammatory agent) [91]. Moreover, Braga et al. [92] reported the in vitro inhibition of the release of human neutrophil elastase. Moreover, Liang et al. [93] showed that thyme inhibited the production of interleukin (IL)-6 and tumor necrosis factor-alpha in mouse cells and suppressed the expression of cytochrome C oxidase-2.

Ocaña and Reglero [94] studied three *Thymus* spp. with concentrations of thymol ranging from 10.05 to 71.15%, and these authors concluded that thymol reduced the gene expression of tumor necrosis factor-alpha, IL-1B and IL-6 in macrophages.

Thymol also plays a role in the antimicrobial activity of thyme EO and has been reported to show an antiseptic property that is 30 times higher than that of other phenol compounds. These components show antifungal and antibacterial bioactivity against both pathogenic microflora and food spoilage microflora.

Other studies have shown the antimicrobial properties of thyme essential oil against the Gram-negative bacterium *Erwinia amylovora*, such as the study of Karami-Osboo [95]. Moreover, it has been reported to have an antimicrobial effect against *Colletotrichum gloeosporioides* and *Rhizopus stolonifera* [96]. *Thymus* sp. showed strong antifungal activity against the pathogens *Penicillium italicum*, *Penicillium digitatum* and *Botrytis cinerea* [97]. In this same line, Satya et al. [98] reported an inhibitory effect against *C. albicans* and *A. niger*. The main compound contained in the oil with anti-fungicidal activity is camphor.

Thymus vulgaris extract has also shown antibacterial activity against *S. epidermidis*, *E. coli*, *Micrococcus mirabilis*, *B. subtilis*, *S. typhimurium*, *Enterobacter cloacae*, *S. aureus*, *S. enteritidis* and *P. aeruginosa* [99].

## 7. Food Applications

Since ancient times, herbs have been used to improve the look and taste of food; however, herbs and spices can also decrease the use of other unhealthy ingredients, such as synthetic additives (such as glutamate, synthetic antioxidants and antimicrobials), sugar, fat and salt.

The use of thyme in food is limited almost entirely to meat products, where it is used for technological purposes, mainly as an antioxidant and preservative. The literature published so far demonstrates that the direct addition of natural antioxidants from thyme can inhibit the development of rancidity in different types of foods. In this context, numerous studies have been carried out that have reported the preservative properties of thyme.

The features of the food (to which thyme is added) can influence the bioactivity of compounds and their preservative properties in foods; such factors include storage conditions, composition and the types of microorganisms in the food. All of these aspects are related to their preservative properties [100]. In addition, the presence of interfering compounds, such as antioxidants, preservatives, additives and nutrients in foods, can reduce the bioactivity of the compounds [100].

The proximate composition of food is very important, and several interactions have been described between protein and phenolic compounds that reduce the action of the bioactive compound; similarly, fat content was reported to have a protective effect on bacteria [101]. Moreover, the pH of foods has an influence on these compounds because it favors the solubilization of hydrophobic essential oils [102].

### 7.1. Sensory Implications

The main objective of the use of thyme in food is to extend the shelf life; however, the main limiting aspect for the use of the essential oil and plant extract of thyme is the development of negative organoleptic characteristics in foods, contributing to an unpleasant odor and taste. To avoid these sensory limitations, several strategies can be employed, such as the use of low concentrations and other conservation methods or the inclusion of natural compounds encapsulated with nanocarriers or added to bioactive films.

On the one hand, the essential oil can be encapsulated with nanocarriers, such as nanoemulsions, nanofibers, cyclodextrins or amylose [100], which mask flavor and contribute to their controlled release while also protecting against oxidative degradation [101]. This strategy increases the bioactivity of compounds present in the EO and plant extract; for example, encapsulation increases the stability of volatile components in the EO and increases cellular uptake, improving antimicrobial activity [102]. Another strategy to decrease the EO concentration is to apply the thyme EO in combination with other antimicrobial and antioxidant compounds to provide synergistic effects without the negative organoleptic aspect. [103].

On the other hand, natural compounds can be included in bioactive films in order to increase the sensory acceptability while also allowing the gradual release of the compound and avoiding the negative organoleptic effects [104].

In spite of the demonstrated potential of thyme and its constituents in vitro, its use as a food preservative has been limited by the organoleptic problem. In many food products, hydrophobic EO compounds are impaired by several interactions with the components of the food matrix, such as fat [105,106], starch [107] and proteins [108]. Moreover, the antimicrobial properties of EO compounds also depend on pH [61], temperature [109] and microbial contamination [109].

Given these interactions between natural extracts and food components, it is useful to study how the constituents of the EO can interact with the food matrix. This interaction can be studied by measuring the microorganism’s growth in a culture medium containing different concentrations of starch, protein and fat.

### 7.2. Incorporation of Thyme in Meat

Different studies have been carried out on thyme to study its properties for stabilizing lipids in meat and meat products. Harpaz et al. [110] reported the effect of the direct addition of thyme EO in commercial foods. In addition, Tanabe et al. [111] studied the use of thyme extract to improve the quality of pork. Medina et al. [112] carried out a study using minced beef, to which they added thyme EO in order to determine its antioxidant power, and as a result of this addition, they reported a decrease in oxidation in the supplemented samples in comparison with control samples. As previously stated, the antimicrobial and antioxidant activity of thyme has been shown in vitro [16] and from its direct application in animal products in general [110,113] as well as veal [114], pork [115] and lamb in particular [116].

To explore the effects of the inclusion of thyme in the animal diet, a study with distilled thyme leaf was conducted by Moñino et al. [116], who investigated whether the introduction of the distilled thyme leaves (10 and 20%) to the feed of pregnant sheep could lead to the transfer of phenolic compounds to lamb meat by studying the lipid stability of the meat (by using the FRAP, ABTS and DPPH methods) and the antioxidant properties of the thyme leaves. In the muscle deltoideus of lambs from mothers fed distilled thyme leaves, the authors found a higher concentration of ferulic acid, rosmarinic acid, carnosic acid, coumaric acid and carnosol than in control samples. However, this significant increase in phenolic compounds did not mean an improvement in their lipid stability. In contrast, it has been shown in numerous studies that feeding with natural products is a strategy to introduce natural antioxidants into phospholipid membranes, where they can inhibit oxidative mechanisms; for example, the incorporation of thyme (*Thymus vulgaris*) into diet rats contributed to the maintenance of an excellent antioxidant capacity in these animals [55]. Moreover, Botsoglou et al. [114] studied the effect of a thyme diet on oxidation reactions in egg yolk and demonstrated that thyme consumption reduced oxidation in egg yolk.

As previously stated, the beneficial effects of the addition of thyme, both its extract and its essential oil, have been studied in several types of meat: veal [114], lamb [116], chicken [117], pork [118] and beef [119,120,121]. However, there is scarce information on the antioxidant efficacy of thyme from in vivo studies [62]. One of these in vivo studies was conducted by Botsoglou et al. [122] in eggs. These authors evaluated the effect of a hen diet with 3.0% thyme on the oxidative stability of eggs. In this study, it was determined that feeding with thyme reduced the oxidation of egg yolk. Another study was conducted by Tanabe et al. [111], who reported the effective prevention of lipid oxidation when a liquid extract of thyme was incorporated into homogenized samples of swine meat at levels ranging from 0.5% to 2.5% *w*/*w*.

Along the same lines, Ouattara et al. [123] showed that the inclusion of thyme EO at 0.9% did not exert negative effects on the taste and appearance of cooked prawns. In contrast, Kykkidou et al. [113] reported that the inclusion of 0.1% thyme oil in cooked fillets of swordfish vacuum-packed in MA (modified atmosphere) presented a different but sensory acceptable smell, which was positively valued at the sensory level by a panel. These results agree with those shown by Solomakos et al. [121], who reported that beef treated with 0.3% thyme EO was acceptable to panelists; however, it was unacceptable (*p* < 0.05) at the level of 0.9%. Therefore, the administered level affected the acceptability. Along these same lines, previous researchers have reported that high levels of essential oils (which are necessary to achieve bacteriostatic activity against pathogens in food) would not be applicable to food because they result in negative organoleptic properties [16,124].

Other studies have investigated whether the addition of natural extract containing phenolic compounds or inclusion of rosemary or thyme leaves in the diet of pregnant ewes has an influence on the oxidative stability and sensory properties of raw and cooked lamb, poultry, as well as pork and chicken meat [125,126,127,128,129,130,131,132,133,134,135,136]. In one of these studies, the authors reported that the inclusion of thyme in the diet of ewes favored a high antioxidant capacity in samples of lamb meat. Cooked lamb meat supplemented with thyme leaves was observed to have lower levels of TBARS and hexanal, while sensory analysis determined a decrease in smell and rancid taste compared with control samples.

Another form of thyme application is the use of its by-products, such as distilled thyme leaves. These by-products are generated because the thyme essential oil industry generates an excess of waste (distilled thyme leaves) after the distillation of the leaves for the extraction of EO. The effect of supplementation on animal diets with thyme by-products on meat quality was studied by Nieto et al. [126], who investigated the effect of the inclusion of distilled thyme leaves in the diet of pregnant sheep and their effect on the final meat quality of lamb, which was studied during the storage of meat in a MA (modified atmosphere). A total of 36 sheep were randomly divided into three homogeneous groups. One group was fed a basal diet. The diet of the other two groups was modified by replacing 10% and 20% of the basal diet with pellets formed of 50% barley and 50% distilled thyme leaves. In general, the diet supplemented with distilled thyme leaves inhibited lipid oxidation, reducing the content of psychrotrophs and secondary oxidation products (TBARS), while the degree of redness was significantly greater after 7 and 14 days of storage. According to these authors, thyme by-products could be used as a source of natural antioxidants and antimicrobials in sheep feed.

To study the antimicrobial effect of thyme on meat, Hao et al. [125] studied the ability of thyme extract to inhibit the development of *A. hydrophila* and *L. monocytogenes* in cooked and chilled chicken meat. These authors concluded that thyme extract could be useful as an antimicrobial in chicken meat.

Sokmen et al. [82] demonstrated the inhibitory effect of *Thymus spathulifolius* essential oil on 15 species of molds and yeasts, and 9 of the 15 were inhibited. These results coincide with those shown by Lambert et al. [69], Burt [16] and Holley and Patel [124]. For these authors, the combination of an MA and thyme EO resulted in an increase in the shelf life of 9 days as a result of the antimicrobial properties of thyme EO. Similarly, Sing et al. [137] showed that thyme essential oil at a level of 0.5% and 1%, but not at 0.1%, had an antimicrobial effect against *L. monocytogenes* in pork sausages. Ozcan et al. [138] reported that thyme EO at a level of 0.2% did not show antibacterial effects against *Escherichia coli* O157:H7, while at a concentration of 0.2%, it showed higher antimicrobial activity. In addition, Sagdic et al. [139] showed that thyme EO at concentrations of 0.5%, 1%, 1.5% and 2% in a nutrient broth presented a bactericidal effect against *Escherichia coli* O157:H7 that was dependent on the dose.

As previously stated, thymol and carvacrol have been shown to possess in vitro antimicrobial activity against Gram-positive bacteria [68,140,141]. However, fewer studies have shown activity against *Listeria monocytogenes* [68]. Specifically, an in vivo study with thyme EO has shown antimicrobial activity against *Listeria monocytogenes* in minced pork [142]. In addition, Deans and Ritchie [143] evaluated 50 essential oils of plants to study the antimicrobial activity against *Escherichia coli*, *A. hydrophila*, *Yersinia enterocolitica* and *Salmonella pullorum*. Among the oils studied, thyme showed strong antimicrobial activity against *A. hydrophila*. Thymol reduced yeast growth because it caused damage to metabolic and structural enzymes and inhibited the repair mechanisms of heat damage in yeasts [144]. The antifungal activity of the EO of the genus *Thymus* has been reported by numerous studies [77,145,146,147]. Specifically, studies with thyme essential oil have shown that it is an excellent fungal development inhibitor [147,148], and its antimicrobial effect against *Shigella* has been thoroughly shown by Bagamboula et al. [149].

The effect of thymol and carvacrol against *Escherichia coli* has been shown in vitro in several studies [68,150,151]. In contrast, some studies have shown no effect or very low antimicrobial activity by the EO of plants against *Escherichia coli* or *Salmonella* in minced beef [152] or in prepared chicken meat [153]. The effect of thyme on lactic acid bacterial counts (LACs) was studied by Nieto et al. [126] in three groups of pregnant ewes: one group was fed a basal diet (C), and the diets of the other two groups were modified by replacing 10% (TF1) and 20% (TF2) of the basal diet with pellets composed of 50% barley and 50% distilled thyme leaves. On day 21 of storage of lamb meat in a modified atmosphere, the LAC counts for C were 4.00 (log cfu/g), while for TF1 and TF2, they were 3.30 and 3.41 (log cfu/g), respectively. Therefore, the diet with distilled thyme leaves showed a slight antimicrobial effect by reducing LAC counts at the end of storage. The limited antimicrobial effect of distilled thyme leaves on lactic acid bacteria at days 0, 7 and 14 is attributed to the high tolerance of these bacteria to the antimicrobial properties of EO due to their ability to generate ATP and to survive under osmotic stress conditions [124]. This behavior was shown by Kykkidou et al. [113], who studied the effect of thyme essential oil on fillets packaged in an MA and stored at 4 °C. For these authors, thyme had no effect on the reduction of the LAC population in fish. Similarly, Tassou et al. [154] observed that the addition of a mixture of lemon juice/olive oil/oregano oil to fillets packaged in an MA decreased the final LAC counts by 0.5 log cfu/g in comparison with control samples. The observed antimicrobial effect of fresh thyme leaf in these studies coincides with the result of numerous studies on the potential of EOs and their compounds as antimicrobials in food. Studies have been carried out on salads, pate, packaged fresh meat, veal, cooked chicken meat, vacuum-packed ham and mozzarella and other cheeses [125,155,156,157,158,159,160,161]. The inhibitory effect of distilled thyme leaf on microflora, reported in the study of Nieto et al. [126], could be associated with the action of carvacrol and thymol [15,159], which, as explained above, enter the circulatory system and are distributed and maintained in animal tissue [162]. Carvacrol, which is usually used as a food additive for its flavoring properties, also shows a broad spectrum of antimicrobial activities against bacteria, yeasts and fungi [163,164]. The antimicrobial activity of the essential oil present in the fresh thyme leaf is due to the fact that its active components contain hydroxyl groups (–OH), a characteristic that makes them highly antimicrobial. The presence of an aromatic structure with a functional polar group has an influence on the inhibitory properties of EOs. Hydroxyl groups can alter the metabolism of enzymes because they bind to their active sites [160].

The mechanism of inhibition of thyme EO has not been studied in great detail [137], but some aspects are clear, such as the fact that their antimicrobial activity is not due to a specific mechanism but that different phenomena occur with several cellular targets [158,165]. Not all of these mechanisms work individually: some of them are the consequence of others [17]. An important characteristic of EOs is their hydrophobicity, which allows them to break the lipid chains of the bacterial and mitochondrial cell membrane, disrupting their structures and making them more permeable [166], and after this process, cellular contents and ions leak from the cell [136]. Therefore, the substantial loss of ions, cell membranes and critical molecules leads to death [167]. In addition, phenolic compounds are able to interact with protein membranes, causing deformation in their structure and interfering with their functionality [167]. Specifically, Davidson [161] reported that carvacrol and thymol are capable of inactivating enzymes that are essential, reacting with cell membranes and altering genetic material.

The antimicrobial activity of thyme EO has been studied in vitro in model food systems [16,124] and in commercial products such as shellfish [110,168,169], veal [37] and pork [115]. In thyme, the synergy between carvacrol and its precursor (ρ-cimeno) is important for its antimicrobial activity [60]. ρ-Cimeno is introduced to the cell membranes of bacteria to a greater extent than carvacrol, which allows this compound to be transported more easily inside the cell; therefore, a synergistic effect is produced between carvacrol and ρ-cimeno when they are together. Another antimicrobial mechanism of these compounds is that they interfere with the cell membrane (phospholipid bilayer) by increasing its permeability, thereby causing the loss of cellular constituents and inducing energy metabolism problems, impaired nutrient absorption, and electron transport and changes in the synthesis of genetic material [170]. Additionally, Bagamboula et al. [149], who studied the antimicrobial effect of the five major components of thyme EO (thymol, p-cimeno, estragol, carvacrol and linalool) against *S. sonnei* and *S. flexneri*, showed that carvacrol, followed by thymol, demonstrated the greatest antibacterial activity against all microorganisms tested; however, linalool and estragol showed a limited effect.

### 7.3. Incorporation of Thyme in Fish and Seafood

The strategy of using natural extracts as fish and seafood preservative agents is a response to the consumer’s concern about synthetic additives and environmental impacts [171]. In general, for this type of application, both thyme essential oil and extracts are used for immersion (for 30 min, followed by drainage of the product) or applied to films or added to the surface of the fish (on both sides). Different studies on the use of thyme in fish and seafood have applied concentrations ranging from 0.1 to 3%. Another alternative is the inclusion of the EO in coatings and packaging films on seafood to contribute to their preservative effects.

To explore the effects of polymer films containing thyme essential oil, Jouki et al. [172] studied the inclusion of 2% thyme oil in films of quince seed mucilage integrated into refrigerated rainbow trout fillets. These authors reported that thyme inhibited the development of lactic acid bacteria, psychrophilic bacteria, *Pseudomonas* spp. and Enterobacteriaceae.

Moreover, Gómez-Estaca et al. [173] studied the effects of different EOs (including thyme) against 18 bacterial strains in fish muscle extract and concluded that clove oil was the best antimicrobial; the second best was rosemary, followed by thyme and lavender oils. These essential oils were able to inhibit *Pseudomonas fluorescens* and *Pseudomonas aeruginosa*; however, in accord with these results, Huang et al. [174] showed that thyme EO was not able to inhibit *Pseudomonas* spp.

Another food-borne pathogen related to the intake of lightly cooked or raw seafood is *Vibrio parahaemolyticus*. For example, Yano et al. [175] (2006) reported that thyme exhibited antibacterial activity at 30 °C; however, this activity against *Vibrio parahaemolyticus* was weak at low temperatures.

To investigate sensory acceptability, Navarro-Segura et al. [176] studied the inclusion of nanoencapsulated essential oils into cyclodextrin films, and these authors concluded that the use of both strategies (nanoencapsulation and the film) improved the sensory acceptability and decreased microbiological counts and trimethylamine, so the shelf life was extended by 4 days. These authors reported an improvement in the sensory acceptability of seabream stored on ice and packaged with films (containing nanoencapsulated essential oils with cyclodextrin). The lower microbiological counts (Enterobacteriaceae, mesophilic bacteria and psychrophilic bacteria) and trimethylamine allowed the shelf life of the fish to be extended by 4 days. 

In addition, several examples of the use of thyme in fish have been reported, such as the study of Mahmoud et al. [177], who showed that submerging fish fillets (carp) in a 1% thymol/carvacrol solution reduced the initial mesophilic counts, and the shelf life was extended from 4 days to 12 days for fillets stored at 5 °C refrigeration. Similarly, Harpaz et al. [110] observed an increase in the shelf life of perch during its storage for 33 days at 2 °C in comparison with the 12 days of shelf life of the control when the fillets were treated with a mixture of EOs of oregano and thyme at 0.05%. In addition, Ouattara et al. [123] showed that the shelf life of prawns was extended by the use of a combination of thyme oil, γ-irradiation and trans-cinnamaldehyde.

### 7.4. Incorporation of Thyme in Milk

The effects of supplementing animal diets with thyme derivatives and their influence on milk quality were reported by Kraszewski et al. [178]. These authors studied the effects of supplementing the diet of cows with a mixture of aromatic plants containing peppermint (*Mentha piperita* L.), chamomile (*Matricaria chamomilla* L.), nettle (*Urtica dioica* L.), yarrow (*Achillea millefolium* L.) and thyme (*Thymus vulgaris* L.), and they achieved positive results in milk yield and the physicochemical and technological composition of milk. In general, the success of the use of antioxidants in the diet of animals has been confirmed by many authors as a tool to improve the quality of products and derivatives, and their benefits for animal health, although the administration of these antioxidants must be controlled in terms of the quantity and the type of antioxidant administered since frequent use of phenolic compounds in the diet of cows can cause abortion or intoxication [179].

The antioxidant activity of thyme is especially useful in food preservation. For example, the incorporation of *T. zygis* leaves into goat feed resulted in cheeses and milk with added bromatological values, which increased the oxidative stability of a typical cheese with wine [180]. In this case, thyme compounds interfere with oxidation propagation reactions. Table 3 shows a detailed list of foods reformulated with thyme.

## 8. Public Health and Dietary Implications Concerning the Use of Thyme in Foods

The European Commission has accepted different EO components as flavoring in food, such as thymol, eugenol, carvacrol, citral, vanillin, limonene, linalool, carvone and cinnamaldehyde, because they do not present a risk to consumer health. The FDA (United States Food and Drug Administration) classifies these substances as GRAS (generally recognized as safe). Included in this category (essential oils classified as GRAS by FDA) are thyme, clove, cinnamon, oregano, mustard, nutmeg and basil. In addition, in the USA, before an EO can be added to foods, there are regulatory limitations on the accepted daily intake; therefore, before adding an EO, a daily intake survey should be available for evaluation by FDA.

In general, recommendations for the intake of food for healthy eating do not yet include suggested amounts of spices and herbs. Therefore, the recommended intake of spices and herbs should be considered for incorporation into guides for healthy eating in different countries. In addition to the health effects, the use of this plant in foods can be used to totally or partially replace other less desirable ingredients, such as synthetic additives, sugar or salt. The addition of these herbs and spices may be a strategy to stabilize stir-fry dishes, dressings and marinades, casseroles, curries, soups and Mediterranean-style cooking.

Before the application of thyme as a natural extract, several factors must be taken into account, such as the nature of the extracts, fruiting stage, mode of extraction, the concentration of active extract components and the possible synergistic effect between thyme and other components. Thyme extract can be applied as a complementary food preservative in food systems such as milk products, dressings, meat and oils and for the enhancement of functional foods. In addition, other considerations regarding thyme are important and warrant its inclusion in the food industry, such as its non-toxicity, availability and low cost.

## 9. Conclusions

Thyme has been extensively studied for its antioxidant and antimicrobial activities. Currently, new advances and techniques in food technology have facilitated efficient identification, processing and extraction of bioactive compounds from herbs and spices in order to include them in functional foods and nutritional supplements. The strategy to produce bioactive compounds with decreased negative organoleptic characteristics and enhanced shelf life will increase overall applications in foods and will subsequently increase spice consumption and, as a consequence, have a positive impact on human health.

However, just a few essential oils or plant extracts containing phenolic compounds are currently included in foods. These essential oils and natural extracts represent potential replacements of competitive synthetic antioxidants and antimicrobials in food and possible value-added products for human consumption. Nevertheless, there is much to learn in terms of its stability in specific matrices and the relationship between their structures, the biological activity of the bioactive metabolites, synergistic effects and effective doses. These themes are the subject of current and future research. Although more studies are needed to establish the broad spectrum of the health benefits of thyme, recent results are very encouraging. Greater public awareness of the properties and benefits of spices to human health would increase the intake of these functional food products.

In conclusion, the development of functional foods enhanced by the inclusion of thyme with value-added properties is of great interest to the scientific community and to the food industry. Thus, beyond their role in flavor, spices should be considered natural components of our nutrition when added to food.

## Figures and Tables

**Table 1:** Components in thyme leaves (*Thymus zygis*, subsp. *gracilis).*

Compounds	Quantity (%)	
	*Thymus zygis*, subsp. *gracilis*	*Thymus vulgaris* L.
α-Pinene	1.10–3.50	1.9
Camphene	0.40–1.50	1.2
Sabinene	0.10–0.20	-
Myrecene	1.10–2.90	1.1
α-Terpineol	0.50–1.30	0.3
1.8-Cineole	1.70–3.10	2.1
γ-Terpinene	2.90–9.70	5.2
p-Cymene	24.70–40.90	29.1
Linalool	3.50–4.20	3.7
Terpinen-4-ol	1.10–3.50	1.3
α-Terpineol	1.00–3.10	0.3
Caryophyllene Oxide	0.40–1.00	0.5
Thymol	22.30–43.30	38.1
Carvacrol	1.50–2.70	2.3
β-Caryophyllene	-	3.1

Source: [15,19].

**Table 2:** Phytochemical composition of thyme and functional properties of the main components in thyme.

Phytochemical Composition of Thyme	Main Components	Functional Properties	Ref.
*Phenolics acids*	Quercetin	Anti-oxidative polyphenol health benefits: preventive effect against Alzheimer’s disease, anti-infl- ammatory and anti-mutagenic properties.	[22,23,24]
	Ferulic acid		
	Syringic acid		
	Caffeic acid		
	Rosmarinic acid		
	p-cumaric acid		
*Biphenyl compounds*	4,4′-dihydroxy-5,5′-disopropyl-2,2′-dimethylbiphenyl-3,6-dione	Antioxidant activity, deodorant effect	[25,26]
	5,5′-diisopropyl-2,2′- -dimethylbiphenyl-3,4,3′,4/-tetraone		
	4′-hydroxy-5,5′-diisopropyl-2,2′-dimethylbiphenyl-3,4-dione		
*Flavonoids*	Flavonols, Flavones	Antioxidant activity, anti-inflammatory	[25,27]
	Flavonols, Flavone glycosides		
	Methyl flavones, Flavonols		
	Apigenin, Luteolin, Hesperitin		
	Rutin, Quercetin, Hesperidin		
	Kaempferol, Kaempferol-3-O-rutinoside		
*Essential oils*	Limonene	Antioxidant, antimicrobial, antitussive, expectorant, antispasmodic, antibacterial effects	[28,29,30,31,32]
	Linalool		
	γ- Terpinene		
	*p*- Cymene		
	Carvacrol		
	Thymol		

Source: [33].

**Table 3:** Foods reformulated with thyme.

Material and Food	Amount	Model	Results	Ref.
Thyme (essential oil) Bonito fish	880 μL/kg	Application: Mixture in fish patty and stored at 4 °C for 14 days.	During the storage period, peroxide values, total volatile basic nitrogen (TVB-N), and thiobarbituric acid index (TBA-i), were significantly lower in thyme group compared to control group.	[181]
Distilled thyme leaves (*Thymus zygis,* subsp. *gracilis*) Lamb meat	Replacing 10% and 20% of the basal diet of pregnant sheep, with pellets elaborated from 50% barley and 50% distilled thyme leaves.	Application: In vivo. Inclusion of distilled thyme leaves in the diet of pregnant sheep and study their effect on the final meat quality of lamb, which was studied during the storage of meat in a MA (modified atmosphere). A total of 36 sheep were randomly divided into 3 homogeneous groups. One group was fed a basal diet. The diet of the other two groups was modified by distilled thyme leaves.	In general, the diet supplemented with distilled thyme leaves inhibits lipid oxidation and reduced the content of psychotrophs. In contrast, the a* values (redness) was significantly greater in lamb meat treated with thyme, (compared with control meat) at 7 and 14 days of storage.	[126]
Fermented poultry sausage Thyme essential oil	0.25%	Thyme essential oil (Thymus vulgaris) incorporated into fermented poultry sausages for 28 days of ripening.	TBA values were significantly affected by the addition of thyme EO. Decrease on total coliform counts, Enterobacteriaceae counts, and *Staphylococcus aureus* counts.	[182]
Thyme essential oil Grass carp (Ctenopharyngodon idellus)	0.1%	Application by immersion and storage at 4 °C.	Thyme essential oils treatment was found to be effective in delaying lipid oxidation, inhibiting microbial growth, and retarding the increase of K-value, putrescine, TVB-N and hypoxanthine.	[174]
Minced pork	1%	Fresh and dried thyme with and without 1% salt, were mixed in 1% concentration to minced pork (100 g). The meat was stored at 5 °C.	Decreased *E. coli* cell numbers in minced pork with 1 log cfu after 24 h storage at 5 °C.	[183]
Chicken sausages	0.125%	Thyme essential oil incorporated into fresh chicken sausages for 20 days at refrigeration temperature (4 ± 1 °C).	Storage studies revealed that thyme oil (0.125%) incorporated aerobically in packaged and refrigerated fresh chicken sausages had approx. 2–3 days longer shelf life than control. Microbial count of thyme essential oil incorporated products were significantly lower than control and remained well below the permissible limit of fresh meat products (log107 cfu/g). Decrease on TBARS values, total viable, psychrophilic bacteria and yeast and mold counts.	[184]
Thyme or laurel essential oil (1%/each) Bluefish (Pomatomus saltatrix)	0.1%	Stored in ice inoculation thyme on the surface of fish.	Shelf life of treated bluefish with thyme was extended 2 days, compared with control samples. Trimethylamine values and total volatile base nitrogen gave acceptable results for up to 9 days for the control samples and 13 days for samples with thyme. Peroxide values, free fatty acid and thiobarbituric acid were lower for treated samples with thyme than the control. Microbial growth in control samples was significantly higher than treated samples with thyme.	[185]
Distilled thyme leaves (*Thymus zygis,* subsp. *gracilis*) Feeding goats with distilled and non-distilled thyme leaves (*Thymus zygis* subsp. *gracilis*)	One group of goats was fed the basal diet (control), the second and third groups were fed with different levels of distilled (10 and 20%) or non-distilled (3.75 and 7.5%) thyme leaves.	Application: In vivo. Inclusion in goats diet with distilled and non-distilled thyme leaves (*Thymus zygis* subsp. *gracilis*) on the physicochemical composition and technological properties of pasteurized goat milk, and on the physicochemical composition, phenolic content, oxidative stability, microbiology, sensory and texture profile of goat cheese.	Incorporation of *T. zygis* leaves to goats provided cheeses and milks with added bromatological values, which increased the oxidative stability of a typical cheese with wine.	[180]
